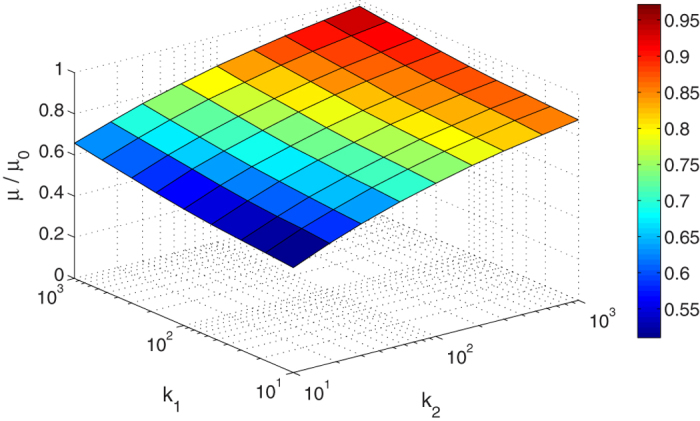# On the role of scales in contact mechanics and friction between elastomers and randomly rough self-affine surfaces

**DOI:** 10.1038/srep11139

**Published:** 2015-06-09

**Authors:** Valentin L. Popov, Andrey Dimaki, Sergey Psakhie, Mikhail Popov

**Affiliations:** 1 Berlin University of Technology, Berlin, 10623 Germany; 2 National Research Tomsk State University, Tomsk, 634050 Russia; 3 National Research Tomsk Polytechnic University, Tomsk, 634050 Russia; 4 Institute of Strength Physics and Materials Science, RAS, Tomsk, 634021 Russia; 5 Skolkovo Institute of Science and Technology, Skolkovo, 143025 Russia

## Abstract

The paper is devoted to a qualitative analysis of friction of elastomers from the point of view of scales contributing to the force of friction. We argue that – contrary to widespread opinion – friction between a randomly rough self-affine fractal surface and an elastomer is *not* a multiscale phenomenon, but is governed mostly by the interplay of only *two* scales – as a rule the largest and the smallest scales of roughness of the contacting bodies. The hypothesis of two-scale character of elastomer friction is illustrated by computer simulations in the framework of the paradigm of Greenwood, Tabor and Grosch using a simplified one-dimensional model.

In practical analysis of engineered systems, the simplest friction law of Amontons^1^ is usually used, stating that the force of friction, *F*, is proportional to the normal force, *F*_*N*_: *F* = μ*F*_*N*_, the proportionality coefficient μ being called coefficient of friction. It is no secret that Amontons’ “law” is only a very rough zeroth-order approximation of real friction. Already Coulomb knew that the coefficient of sliding friction depends on sliding velocity and normal force and that static friction depends explicitly on time^2^. However, even 230 years after Coulomb no generalized laws of friction exist that could be reliably used in engineering practice. It is often argued that the reason for this lies in the multi-scale nature of friction, and that *all* scales necessarily have to be included into consideration to achieve a realistic model of friction. In the present paper we will argue that this is not the case: In friction there exist characteristic scales which provide the main contribution to the quantities of interest. Therefore, as in most areas of physics, the most productive approach is searching for the relevant scales and studying them. The present work is devoted to an analysis of the contribution to friction of different scales for one class of materials – elastomers with linear rheology.

## The roles of micro- and macro-scales in elastomer friction

**Figure 1:**
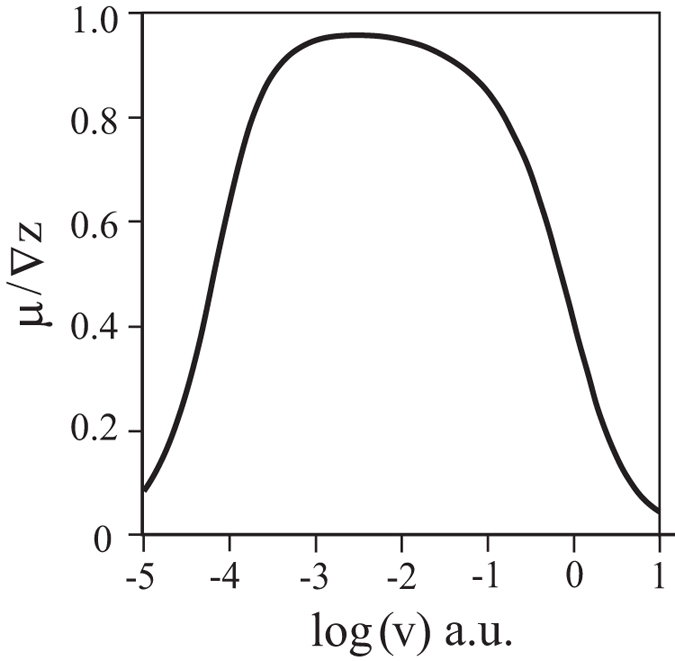


**Figure 2:**
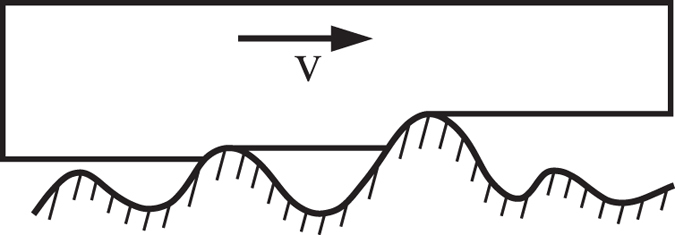


Since Greenwood and Tabor^3^, and especially after the classic work of Grosch^4^, it has been widely accepted that elastomer friction is mostly due to internal dissipative losses in the material that are caused by deformation through surface asperities of the counter body. In the present paper, we follow the above paradigm of Greenwood-Tabor-Grosch and do not discuss the adhesive contribution to friction. The force of friction typically increases with velocity, reaches a plateau and decreases again, as schematically shown in Fig. 1. The plateau is normally of most practical interest. Physically, the behavior of an elastomer in this range is dominated by the loss modulus of the elastomer^5^, and the elastomer behaves roughly speaking as a simple fluid. In particular, the relaxation of the elastomer after indentation or ploughing by an asperity is very slow. Practically all micro contacts therefore will be in one-sided contacts as shown in Fig. 2. It is easy to understand that the coefficient of friction is then roughly equal to the one-sided average of the local slope of the surface profile in the contact region. This simplified picture is valid if the contact between elastomer and the rigid body is friction free on scales smaller than that of the asperities.

Despite the apparent simplicity of this physical picture, already at this point an interesting and non-trivial question arises: What is the average slope of the profile? Let us start the discussion of this question with an estimation of the rms value of the gradient of the surface profile over the whole surface. For illustration, we consider the simplest case of the so-called “randomly rough” surfaces, which can be completely characterized by the *spectral power density C*(*q*) of the surfaces profile, (definition see e.g. in^6^). For this type of surface topography, the average gradient of the surface profile, ∇*z*, can be expressed as an integral of the spectral power density according to
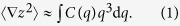


It is known that many natural surfaces have the property of fractality, at least in a limited range of wave vectors (these “limited” fractal surfaces are sometimes called “natural” or “physical” fractals, see, e.g.^7^). In this limited range, the spectral power density of typical fractal surfaces is known to be a power-function of the wave-vector *q*:
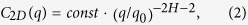
where *H* is the Hurst-Exponent and *q*_0_ is some reference wave-vector^8^. For Hurst exponents smaller than one, the resulting integral diverges at the upper limit of integration. This means that for a *true fractal surface* (without an upper cut-off wave vector), the surface gradient would be infinitely large. In practice, of course, there is always some upper cut-off wave vector *q*_max_, and the surface gradient is determined by one or two orders of magnitude of wave vectors at and below *q*_max_. In other words, *for typical fractal rough surfaces, the friction force is determined by the roughness components with the largest wave-vectors (or the smallest scale of the system).* One can say that understanding friction is equivalent to understanding the nature of this smallest relevant scale. The conclusion that the surface gradient is mainly determined by the smallest space scales follows from very general scaling considerations of self-affine fractals and is not limited to the spectral representation of rough surfaces. However, for the sake of simplicity and transparency of argumentation, we will confine ourselves to consideration of randomly rough surfaces according to definition^6^.

Of course, the above estimation is oversimplified in the sense that it is the surface slope *in the contact region* and not over the whole surface which is determining the coefficient of friction at the plateau. However, as has been shown already by Archard in 1950th^9^, the main effect of changing normal force is the number of asperities coming into contact, while the local conditions in the real contact area, including the surface gradient, depend only weakly on the normal force. Thus, the average gradient over the whole surface is already a good estimation for the gradient in the real contact area. However, the mentioned relatively weak dependence on the normal force is exactly what we would like to discuss in more detail.

The actual surface gradient is a function of the current contact configuration (e.g.,^10^). In the next section we will argue that the governing parameter for the contact configuration is the *indentation depth d.* The indentation depth, in turn is connected with the normal force over the contact stiffness, which is dependent practically only on the large wavelength components of roughness (or on the macroscopic form of the indenter)^11^. We will thus come to the following hypothesis: *while the friction force is almost entirely dependent on the smallest-scale roughness, its weak dependence on the normal force is related only to the large scale roughness*. We then will substantiate this *two-scale hypothesis* with a numerical simulation of the force of friction between an elastomer and a randomly rough fractal surface using a simplified one-dimensional model.

## Indentation depth as a governing parameter of contact configuration

If a rigid body of an arbitrary shape is pressed against a homogeneous elastic half-space then the resulting contact configuration is only a function of the indentation depth *d*. At a given indentation depth, the contact configuration does not depend on the elastic properties of the medium, and will be the same even for indentation of a viscous fluid or of any linearly viscoelastic material. This general behavior was recognized by Lee^12^ and Radok^13^ and was verified numerically for fractal rough surfaces^14^. Further, the contact configuration at a given depth remains approximately invariant for media with thin coatings^15^ and for multi-layered systems, provided the difference of elastic properties of the different layers is not too large^16^. In^17^, it was argued that this is equally valid for media which are heterogeneous in the lateral direction (along the contact plane). Along with the contact configuration, all contact properties including the real contact area, the contact length, the contact stiffness, as well as the rms value of the surface gradient in the contact area will be unambiguous functions of the indentation depth. The indentation depth is thus a convenient and robust “governing parameter” for contact and frictional properties of media with linear rheology. Note, that this is equally valid for tangential contact. This can easily be illustrated with the example of contact of a rigid body with an incompressible elastic half-space: For a circular contact with an arbitrary radius *a*, the ratio of the normal stiffness *k*_*z*_ and the tangential stiffness *k*_*x*_ is constant and given by the Cattaneo-Mindlin factor^18^,^19^ for incompressible media *k*_*z*_/*k*_*x*_ = 1.5. From this follows that for a frictional contact with the coefficient of friction μ, the maximum tangential displacement to the onset of complete sliding is determined solely by the indentation depth and is equal to *u*_*x*,max_ = 1.5 μ*d*^20^,^21^. This result does not depend on the form of the body and is valid for arbitrary bodies of revolution and even for randomly rough fractal surfaces^22^.

In practice, however, the controlled and measured quantity is normally not the indentation depth but the normal force. The latter is connected with the indentation depth through the *contact stiffness*. The contact stiffness, however, is known to be determined almost entirely by the long wave-length part of the spectral density of the surface profile (or the macroscopic form of the contacting bodies) and does not depend on the detailed topography on the micro-scale^11^. This supports the hypothesis that frictional force is determined mainly by the smallest spatial scale while its weak dependence on the normal force is governed almost exclusively by the largest spatial scale of the system.

## Numerical simulation of a frictional contact

Despite the simplicity and robustness of the arguments for the two-scale picture of friction and contact of rough surfaces, they are not exact. It is therefore of interest to test by direct simulation if the arguments are valid and with what accuracy. The main statement of the “two-scale” concept is that the frictional and contact mechanical properties do depend only on the upper and lower parts of power spectrum of the rough surface and are practically not sensitive to the middle part of the spectrum. We therefore performed numerical simulation of the contact of a rigid fractal surface using the full fractal spectrum and a spectrum with a truncated middle part. Note that we use the fractal approach (as described e.g. in^23^) for generation of the rough surface profile (instead of the Cantor^24^,^25^ or Weierstrass^26^ approaches, for instance). This is done for illustrative purposes, as the randomly rough surfaces can be easily handled numerically in the frame of this approach. However, we beleve that the main qualitative arguments of the paper remain valid also for other types of fractal surfaces.

We considered the simplest contact of rigid rough surfaces with a viscoelastic (Kelvin) counter-body. According to the general logic presented in the introductory sections, we wanted to check the following properties:

1. The indentation depth is the governing parameter of the contact. Thus, the length of the system under consideration (which determines the minimal wave-vector of the fractal surface) does not influence the coefficient of friction, provided the indentation depth is kept constant.
2. Under conditions of constant normal force, the length of the system (and, thus, the minimal wave-vector) does influence the coefficient of friction.
3. The coefficient of friction is influenced mostly by the short wavelength part of power spectrum and is insensitive to changes in the middle part of the power spectrum.

**Figure 3:**
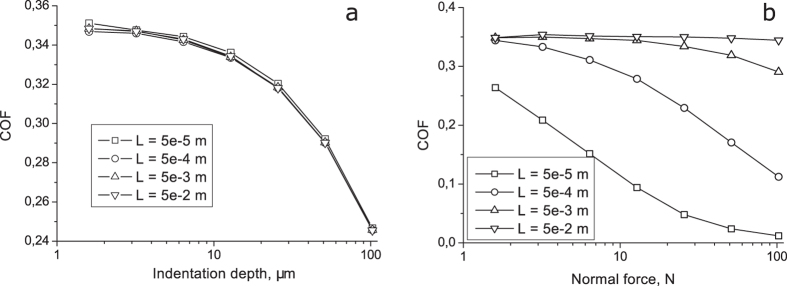
Dependencies of the coefficient of friction on indentation depth (**a**) and normal load (**b**) for the surfaces of different length L.

To test the first property, we calculated the coefficient of friction for systems of different total length (and thus different long wavelength cut-off wave-vector, while the upper part of the spectrum remained unchanged). The results are presented in Fig. 3a. They show that under conditions of constant indentation depth the coefficient of friction practically does not depend on the length of the system. However, under conditions of controlled normal force, there is distinct dependency on the size of the system, as it should be, Fig. 3b. The reason is that the size of the system influences the contact stiffness and thus the indentation depth for a given normal force. We would like to stress that in spite of the force dependence of the “coefficient of friction”, we still prefer to use this quantity (formally defined as the ratio of the tangential and normal force), as it has simpler physical properties than the frictional force itself.

**Figure 4:**
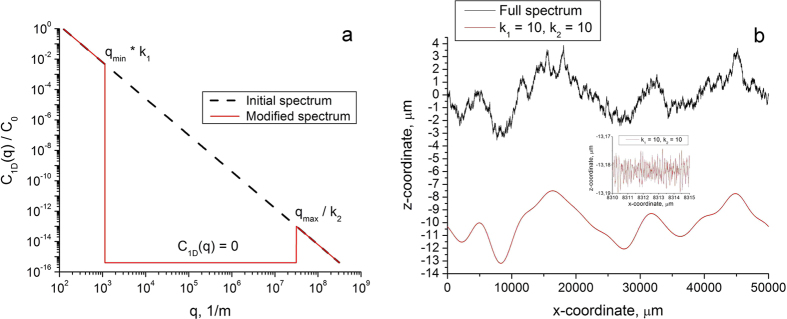
(**a**) Spectral density of a fractal surface. The black dashed line corresponds to the initial “full fractal” spectrum. The red solid line shows a modified spectral density. By changing the coefficients *k*_1_ and *k*_2_ one can change the size of the truncated region. (**b**) Rough profile generated with the complete power density (upper curve) and truncated power spectrum. In the second case, the curve loses its “fractal character”, which however, has practically no effect on the coefficient of friction.

The central point of the two-scale picture of rough contacts is the insensitivity of contact properties to the middle part of the power spectrum. To show this, simulations were carried out first with the complete fractal power spectrum given by equation (2) as shown with a dashed line in Fig. 4. The spectrum then was modified by setting equal to zero the middle part of the spectrum as shown in Fig. 4 with red solid line. The coefficients *k*_1_ and *k*_2_ determine the size of the inner truncated region of the spectral density.

**Figure 5:**
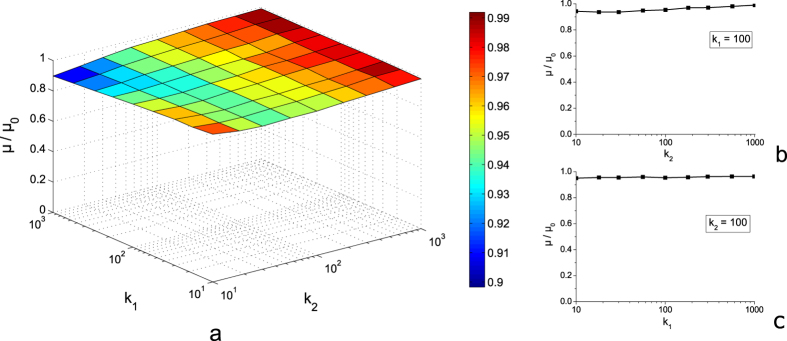
The dependence of coefficient of friction on the values of *k*_1_ and *k*_2_ for *H* = 0.5 (**a**); cross-sections of this dependence at *k*_1_ = 100 (**b**) and *k*_2_ = 100 (**c**).

We used roughness with the ratio of *q*_max_/*q*_min_ ≈ 10^6^. This allowed studying the influence of the coefficients *k*_1_ and *k*_2_ in a wide range of their values. Results for different indentation depths were very similar, we therefore present below only results for large indentation depth *d* ≈ 6 *h* (*h* is the rms value of roughness) providing complete contact. Velocity was chosen so that the coefficient of friction would correspond to the beginning part of the “plateau” region. Figure 5a shows that for surfaces with the Hurst exponent *H* = 0.5 removing large parts of the spectral density corresponding to the range of values of *k*_1_∈[10; 1000] and *k*_2_∈[10; 1000] practically does not change the value of the coefficient of friction. The deviation of the coefficient of friction for a modified surface from its initial value doesn’t exceed 10 percent.

Note that the two-scale property of friction becomes less precise for large Hurst exponents *H* ≈ 1. For *H* = 1, the integral (1) diverges logarithmically both on the lower and the upper limit of integration. Only in this limiting case, the friction is truly multi-scale, and all parts of power spectrum contribute essentially to the coefficient of friction. To illustrate this special case, we carried out simulations for roughness with Hurst exponent *H* = 0.9. The results shown in Fig. 6 demonstrate that in this case the coefficient of friction shows a significant dependency on the size of the truncated region of the spectral density. However, such high Hurst exponents are not typical for frictional surfaces.

## Discussion and conclusions

We have shown that the multi-scale view of friction adds very little to the accuracy of friction models – at least for the case of elastomer friction – while making them significantly more complicated. The most important processes in friction happen at two distinct scales – the largest and the smallest one. Therefore, any model that correctly describes the contact stiffness on the largest scale and reproduces the gradient of the surface profile on the smallest scale, is eligible for the study of frictional processes (examples of models based on the two-scale nature of frictional contacts can be found in^27^,^28^). The same is valid for other properties such as contact area, contact stiffness, adhesion force, electrical conductivity and so on. Our main line of reasoning is not based on any particular representation of rough surfaces. As a matter of fact, only the following statements are essential to our argumentation:

1. According to Grosch, at least in the region of plateau of the dependency of the coefficient of friction on velocity, it is the *surface gradient* which determines the coefficient of friction.
2. The surfaces usually have larger surface gradient on smaller scales, thus, usually the *smallest scales give the main contribution to the coefficient of friction*.
3. The *governing parameter of the contact configuration is the indentation depth*, and the latter is connected with the normal force through stiffness, which is determined practically only by the macroscopic form or the largest wave length contributions to the roughness.

All these key points are more or less model independent.

Our conclusion seems to contradict the recent conclusion of Barber^29^ that Amontons’ law is a direct consequence of the multiscale nature of most rough surfaces. In reality, however, there is no contradiction: the authors completely agree with the arguments and results of Barber and see only a terminological confusion. It is worth discussing it briefly to avoid misunderstandings. The paper of Barber is devoted to the discussion of the reasons of the (approximate) validity of Amontons’ law of friction. The main conclusion of it is that «Amontons’ law of friction follows inevitably from the multiscale character of rough surfaces, provided only that in regions of actual contact there exists some (but arbitrary) relation between the local normal pressure and the shear traction during sliding». This conclusion coincides with results reported by Popov *et al.* in^21^, Chapter 10 and by Pohrt in 8. Note that Barber classifies as multiscale all classical theories of rough surfaces (e.g. those of Archard, Greenwood and Williamson^30^ etc). His definition of the “multiscale property” is just the existence of some sufficiently broad distribution of heights of asperities – independently of other properties such as their form, interaction or local friction law. However, other authors classify the Greenwood-Williamson theory as a one-scale theory and claim that due to this property it cannot be correct. In our analysis we use the following definition of “one-scale roughness”: roughness of one scale is composed by components of spectral density spreading no more than about one order of magnitude around a given wave vector. According to this definition, the Greenwood-Williamson type of surface is one-scale roughness. Due to the (limited) variance of the wave vector, it is of course still randomly rough and has “sufficient multiscale content” in the sense of Barber’s paper while still possessing a characteristic wave vector and not representing a true multiscale or fractal surface.

## Methods

For numerical simulations of friction, we used the one-dimensional model of a contact between an elastomer and a randomly rough fractal surface described in^27^. In this model, the elastomer is represented as a one-dimensional series of Kelvin elements with parameters determined by the rules of the method of dimensionality reduction (MDR),^21^. We considered an elastomer with elastic modulus *E* = 10^7^ *Pa* and viscosity of η = 10^7^ *Pa*·s. The spatial step of simulated system was Δ*x* = 10^−8^ m, the length of the system was varied from *L* = 5·10^−5^ m to *L* = 5·10^−2^ m. The spectral density of the surface was determined in accordance with equation (2), with Hurst exponent of *H* = 0.5. The lower cut-off wave vector was determined by the length of the system *q*_min_ = 2π/*L* and the upper cut-off wave vector by the spacing Δ*x*: *q*_max_ = π/Δ*x*.

## Additional Information

**How to cite this article**: Popov, V. L. *et al.* On the role of scales in contact mechanics and friction between elastomers and randomly rough self-affine surfaces. *Sci. Rep.* **5**, 11139; doi: 10.1038/srep11139 (2015).

## Figures and Tables

**Figure 6:**